# Fourier Transform Infrared Spectroscopy Reveals Molecular Changes in Blood Vessels of Rats Treated with Pentadecapeptide BPC 157

**DOI:** 10.3390/biomedicines10123130

**Published:** 2022-12-04

**Authors:** Ozren Gamulin, Katarina Oroz, Luka Coric, Maria Krajacic, Marko Skrabic, Vilim Dretar, Sanja Strbe, Jasminka Talapko, Martina Juzbasic, Ivan Krezic, Marin Lozic, Vasilije Stambolija, Helena Zizek, Ivana Jurca, Ivana Jurjevic, Alenka Boban Blagaic, Anita Skrtic, Sven Seiwerth, Predrag Sikiric

**Affiliations:** 1 Department of Physics and Biophysics, School of Medicine, University of Zagreb, 10000 Zagreb, Croatia; 2 Department of Pharmacology, School of Medicine, University of Zagreb, 10000 Zagreb, Croatia; 3 Faculty of Dental Medicine and Health, Josip Juraj Strossmayer University of Osijek, 31000 Osijek, Croatia; 4 Department of Pediatric and Preventive Dentistry, School of Dental Medicine, University of Zagreb, 10000 Zagreb, Croatia; 5 Department of Anesthesiology, Resuscitation and Intensive Care, University Hospital Centre Zagreb, 10000 Zagreb, Croatia; 6 Department of Pathology, School of Medicine, University of Zagreb, 10000 Zagreb, Croatia

**Keywords:** stable gastric pentadecapeptide BPC 157, Fourier transform infrared spectroscopy, vessel

## Abstract

Recently, it was found that when confronted with major vessel occlusion and vascular failure, stable gastric pentadecapeptide BPC 157 therapy might rapidly functionally improve minor vessels to take over the function of disabled major vessels, reorganize blood flow, and compensate failed vessel function. We focused on the BPC 157 therapy effect obtained by giving 10 ng/kg ip to rats 5 min before sacrifice on the rat thoracic aorta, which we assessed with Fourier transform infrared spectroscopy (FTIR) 90 min thereafter. We applied a principal component analysis (PCA). The PCA model showed, with a clear distinction being mostly due to the PC1 score, differences between the spectra of BPC 157- and saline-treated rats. The comparison of the averaged spectra of these two groups with their differential spectrum and PC loadings allowed us to identify the parts of the FTIR spectra that contributed the most to the spectral separation of the two observed groups. The PC1 loadings and the differential spectrum showed that the main bands affecting the separation were the amid I band around 1650 cm^−1^, the amid II band around 1540 cm^−1^, and the vibrational band around 1744 cm^−1^. Fitting the spectral range between 1450 and 1800 cm^−1^ showed changes in protein conformation and confirmed the appearance of the vibrational band at 1744 cm^−1^. Controls had a substantially more intense vibrational band at 1744 cm^−1^. These spectral results showed the cells from saline-treated (control) rats to be in the early stage of cell death, while the samples from BPC 157-rats were protected. Thus, BPC 157 therapy changed the lipid contents and protein secondary structure conformation, with a rapid effect on vessels, within a short time upon application.

## 1. Introduction

We focused on the cytoprotective stable gastric pentadecapeptide BPC 157 [1,2,3,4] effect on the rat aorta, which we assessed with Fourier transform infrared spectroscopy (FTIR). Recently, some particular stable gastric pentadecapeptide BPC 157 therapy effects have been especially reviewed, including prompt beneficial vascular effects; the particular activation of the collateral pathways to recover severe vessel and multiorgan failure occlusion/occlusion-like syndrome; the recovery of muscle disturbances; and striated, smooth, and heart failure recovery as a whole [1,2,3,4]. In addition, BPC 157 might particularly interact with nitric oxide (NO) and prostaglandin systems [5,6], maintain thrombocyte function [7,8,9], act as a membrane stabilizer (counteracting leaky gut) [10], and act as a free radical scavenger, particularly in vascular studies [10,11,12,13,14,15,16,17,18]. Now, to reveal the background of the recovering vascular effect with BPC 157 therapy immediate application, this report describes the vibrational status of the vessel tissue components as the essential beneficial changes that might be important for rapidly emerging beneficial effects on endothelium and blood vessel syndromes [12,13,14,15,16,17,18,19,20,21,22,23,24,25,26]. Thus, these might be essential for the described particular vascular therapeutic effects [12,13,14,15,16,17,18,19,20,21,22,23,24,25,26] and a novel indicative resolving key.

The vibrational status of tissue components might be important due to the particular effect on the endothelium and blood vessels that BPC 157 might have as an original cytoprotective agent [4]. Noteworthily, in the 1980s, upon Robert’s original demonstration of the strong prevention of intragastric alcohol-induced stomach necrosis (cytoprotection and direct cell protection) [27], as essential, additional initial effects, cytoprotective agents were shown to commonly protect the endothelium [28,29]. Therefore, cytoprotective agents might have a pleiotropic beneficial effect, but standard cytoprotective agents still have considerable limitations in practical therapeutic applications [27,28,29]. Nevertheless, given that direct endothelium cell protection has been described to occur in the stomach within less than 30 s [28,29], the stable gastric pentadecapeptide BPC 157, as peptide that is native to and stable in human gastric juice, seems to be fully inclined to innate cytoprotection function, the continuous maintenance of the stomach and gastrointestinal mucosa, and endothelium integrity [1,2,3,4]. Therefore, as a novel cytoprotective mediator with high wound-healing capabilities [1,2,3,4,5,30], it provides greater pleiotropic cytoprotection beneficial potential and effects, which might be easily applicable in practical therapy [1,2,3,4,5]. There might be a particular way for the stable gastric pentadecapeptide BPC 157 and its effect, to accordingly combine together, the functionally improved Robert’s and Szabo’s original maxim endothelium maintenance → epithelium maintenance [27,28,29], and rapidly and functionally improved minor vessels. This might be to take over the function of disabled major vessels, reorganize blood flow, and compensate failed vessel function [1,2,3,4,5]. Illustratively, the azygos vein (superior–inferior caval vein shunt) could provide more direct blood flow delivery [1,2,3,4,5,12,13,14,15,16,17,18,19,20,21,22,23,24,25,26]. This might result (confronted with major vessel occlusion; peripheral, central, or alike noxious procedures; occlusion/occlusion-like syndrome) in the particular resolving activation of collateral pathways related to the given injury [1]. Many examples of severe noxious events have been reported, all as progressing Virchow triad circumstances [12,13,14,15,16,17,18,19,20,21,22,23,24,25,26]. Specifically, to counteract the adverse effects tightly combined in occlusion/occlusion-like syndrome, there are various options, such as the occluded superior mesenteric artery and/or vein [14,15,16], the inferior caval vein, the infrarenal or suprahepatic vein [12,20], the superior sagittal sinus [24], and the episcleral veins [19]; these can also be employed in alike noxious procedures [21,22,23,24,25], maintained intra-abdominal hypertension [21], diverse noxious agents’ application [23,24], myocardial infarction [22], and acute pancreatitis [25]. Given the large blood volume commonly trapped with(in) failed/occluded vessels [12,13,14,15,16,17,18,19,20,21,22,23,24,25,26], these vascular activating effects occur as specific novel rapid therapeutic effects [1,2,3,4,5] against confronted multiorgan failure due to severe vascular failure [1,2,3,4,5], which may be generally seen peripherally and centrally [12,13,14,15,16,17,18,19,20,21,22,23,24,25,26] as overwhelming severe syndrome. Occlusion/occlusion-like syndrome presents with the severe intracranial (superior sagittal sinus) hypertension, portal and caval hypertension, and aortic hypotension, and these can be attenuated/eliminated with BPC 157 therapy [12,13,14,15,16,17,18,19,20,21,22,23,24,25,26]. Consistently, with BPC 157 therapy, it has been reported that the following can be counteracted: lesions and hemorrhage in the brain and heart (congestion, infarction, and arrhythmias); congestion and hemorrhage in the lung, liver, and kidney; gastrointestinal lesions; and muscle weakness. In particular, progressing thrombosis can be almost annihilated peripherally and centrally. Virchow triad circumstances can be recovered, as previously reported [12,13,14,15,16,17,18,19,20,21,22,23,24,25,26]. Blood vessel failure (congested (i.e., inferior caval vein and superior mesenteric vein) and collapsed (azygos vein)) can be reversed to normal vessel presentation [26]. In eye surgery inducing glaucoma (cauterization of three of the four episcleral veins), the remaining episcleral vein, when functionally improved with BPC 157 therapy, might substitute the function of the other episcleral veins [19]. So, BPC 157 therapy might counteract increased intraocular pressure, retinal ischemia, and glaucoma development and might reverse already advanced glaucoma course [19,31]. Finally, this vascular effect might have equal strength in BPC 157 therapy under ischemic conditions as in therapy started later in advanced reperfusion [13,18,32,33,34].

Likely, this therapeutic effect [12,13,14,15,16,17,18,19,20,21,22,23,24,25,26] might present as an effect on the whole blood vessel wall. Conceptually, this functional improvement effect on blood vessels might be achieved immediately upon administration; then, it might be sustained to ascertain adequate blood vessel function to prevent and/or reverse the otherwise inevitable adverse effects that might be created by upcoming noxious events [12,13,14,15,16,17,18,19,20,21,22,23,24,25,26]. Noteworthily, the cytoprotection concept holds that the direct effect on blood vessels occurs as an essential cytoprotective effect that further maintains tissue integrity even under ex vivo conditions [4,5]. Thus, to substantiate this essential, rapid vascular effect, we used FTIR to discriminate particular cell components; therefore, the assessment was performed using thoracic aortas obtained from rats that received BPC 157 or saline therapy shortly before sacrifice. As a particular novelty point, it appears that the changes in aortic tissues under the influence of any drug with infrared spectroscopy have not been investigated so far. FTIR spectroscopy [35] involves the measurement of many spectra of a sample, each containing the chemical signatures (absorption bands related to vibrational modes of specific chemical bonds) of compounds at a specific location within the specimen. We recorded the whole mid-range infrared spectrum (600–3900 cm^−1^). To show the spectral differences, we used a principal component analysis (PCA). Moreover, the differences were the most pronounced in the lipid part of the spectrum.

## 2. Materials and Methods

### 2.1. Tissue Samples

We compared the spectra of abdominal aortic tissue samples from 10 Wistar Albino male rats, 200 g in body weight, either treated with BPC 157 (10 ng/kg ip) or treated with saline (5 mL/kg ip) 5 min before sacrifice. Then, abdominal aortic tissues were embedded in distilled water before further procedures (for 5 min). From each rat (5 rats per group), 20 samples were cut, and the tissue samples were put in silicon windows. Then, the samples were dehydrated (removing excess water from tissue, not from cells) under vacuum, since FTIR spectra are sensitive to water. Subsequently, the FTIR spectra of the samples were recorded. Ninety minutes was the time elapsed between the sacrifice and FTIR spectrum recording. Note that all sections were 10 to 15 µm thick. Each of the 20 tissue sections from every rat became a sample for FTIR spectroscopy.

### 2.2. FTIR Spectroscopy

The vibrational spectra of the samples were recorded with a Perkin-Elmer Spectrum GX spectrometer equipped with a liquid N_2_ refrigerated Mercury Cadmium Telluride (MCT) detector. Optical-grade silicon windows were used for acquiring the 1000-scan background, which was automatically subtracted from the tissue spectra. One hundred scans were recorded for each tissue section to obtain the vibrational spectra, which lasted around 5 min for each sample. Data were acquired in the 450–4000 cm^−1^ spectral range, in transmission mode, with a resolution of 4 cm^−1^. A sample area with a diameter of approximately 1 cm was recorded. The resulting spectrum was a sum of the contributions of all the tissues in the sample area with characteristics depending on the relative contributions of those tissues (i.e., different relative concentrations of standard molecules existing in every biological material).

### 2.3. Data Analysis

Matlab10 (Mathworks) was used for processing the spectra. First, all recorded spectra were baseline-corrected and normalized. Baseline correction was performed using the SNIP (statistics-sensitive nonlinear iterative peak-clipping) algorithm. After that, the vector normalization of the spectra was conducted to compensate for spectral differences caused by small variations in the recording conditions. Many physical and chemical factors, such as sample size, humidity, molecular interactions, etc., can affect the recording of spectra. Preprocessing techniques compensate for those deviations and intensify the relationship between the spectral signals and the observed characteristics of tissues.

Matlab10 and PLS Toolbox (Eigenvector Research) were used to perform the principal component analysis (PCA). The PCA is an unsupervised statistical method that reduces the multidimensional experimental data set to a much smaller number of uncorrelated variables called principal components [23]. Usually, only the first two principal components, PC1 and PC2, which account for most of the variance presented in the experimental data, are utilized in the majority of applications. Using PCA, we made a quantitative model, which was used to identify the differences between the control and the treated tissue samples according to their FTIR spectra.

Origin Pro 2019b (OriginLab Corporation, Northampton, MA, USA) was used for the fitting of the vibrational bands.

## 3. Results

**Figure 1:**
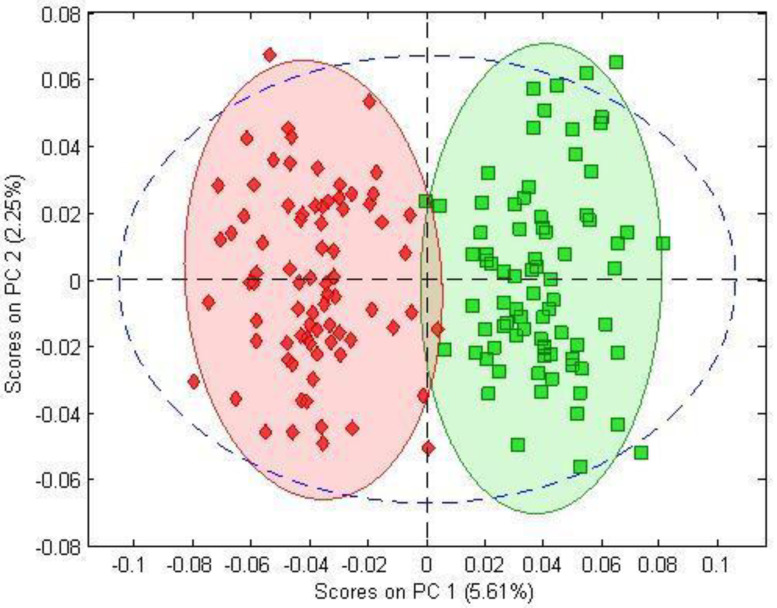
PC1-versus-PC2 score plot resulting from the decomposition of FTIR data with PCA obtained from control samples (red rhombuses) and BPC 157-treated samples (green squares).

The PCA separated the BPC 157-treated and control samples on a PCA score–score graph. Using 100 spectra of both BPC-treated and control samples, the PCA model showed the scatterplot of the first two principal components, with a clear distinction, mostly due to the PC1 score, between the spectra of the BPC 157-treated group (green) and those of the control group (red) (Figure 1).

**Figure 2:**
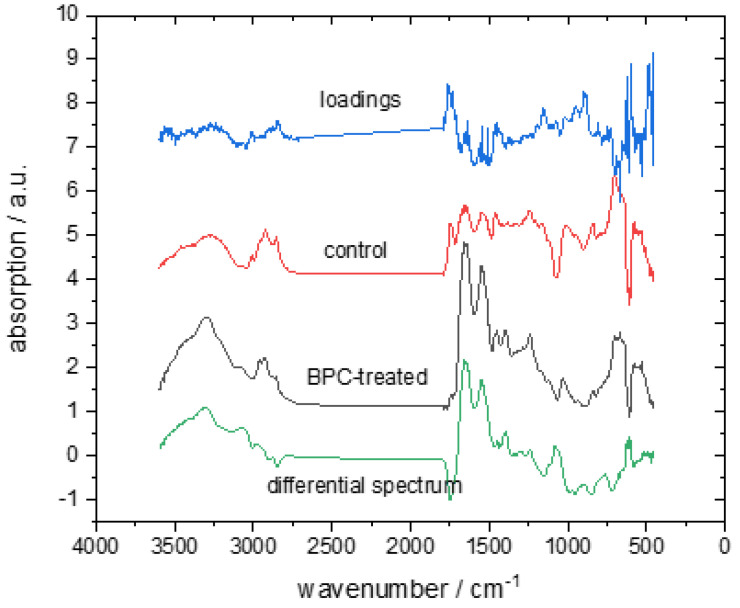
The average FTIR spectra of the control group (red line) and BPC 157-treated (black line) group were obtained in the spectral range between 4000 and 500 cm^−1^. The differential spectrum of the average BPC 157-treated spectrum minus the control spectrum (grey line) and corresponding PC1 loadings (blue line) are shown.

The comparison of the averaged spectra of these two groups with their differential spectrum and PC loadings allowed us to identify the parts of the FTIR spectra that contributed the most to the spectral separation between BPC 157-treated rats and the control group (Figure 2). The two spectra (red and black) in the middle of the figure represent the average spectra of the BPC 157-treated and control groups, respectively. The differential spectrum of the average spectra (BPC-treated spectra minus control spectra) is presented at the bottom of the figure and the PC1 loadings at the top. Only the first principal component was used, because it exhibited the strongest difference between the two observed groups in the analysis of the PCA scores. The samples of the raw FTIR spectra are presented in Figure S1 in the supplementary materials.

The PC1 loadings and the differential spectrum showed that the main bands affecting the separation of the BPC-treated and control sample spectra were the amid I band around 1650 cm^−1^, the amid II band around 1540 cm^−1^, and the vibrational band around 1744 cm^−1^. There was also a small but observable difference in intensity in the spectral range of the CH vibrations of lipids between 2800 and 3000 cm^−1^. Table 1 lists the assignments of vibrational bands relevant to this paper.

**Figure 3:**
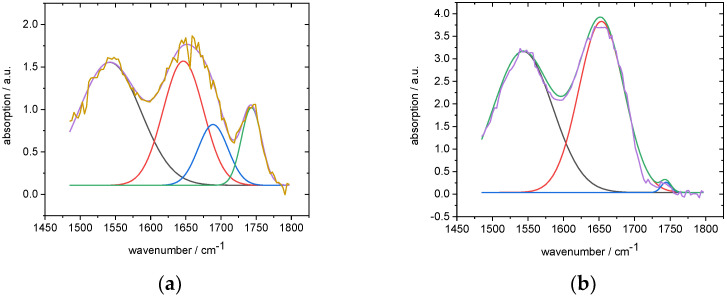
Fitted spectral range from 1450 to 1800 cm^-1^: (**a**) control group (**b**) BPC 157-treated group.

Then, to see the changes in the protein secondary structure, we fitted the spectral range between 1450 and 1800 cm^−1^ with three Gaussians. The amid I band consisted of four vibrational bands related to the secondary protein structure, β-sheet, Random coil, α-helix, and turns (Table 1). However, because of the lower signal-to-noise ratio in the case of the control spectra, we chose an approximate approach with three Gaussians (one each for amide I, amide II, and the band at 1744 cm^−1^) for the BPC 157-treated spectra and four Gaussians for the control spectra (Figure 3). We used the fourth Gaussian for fitting the amid I band of the control spectra. Because the amide I band could not be successfully fitted, the fit did not converge; so, instead of one Gaussian, we had to apply two. The fitting parameters are presented in Table 2.

The fitting results presented differences in the BPC 157-treated and control group spectra (Table 3). The vibrational band at 1744 cm^−1^ was substantially more intense in the control group. Changes in the protein secondary structure were also discovered. The average position of the amid II band in the control group was shifted toward lower wavenumbers, and the amid I vibrational band had to be fitted with two Gaussians instead of one, as in the case of the BPC 157-treated samples. The intensity values of the amid I and amid II vibrational bands were much lower in the control group spectra. Those changes in the amid I and amid II bands indicated changes in the protein secondary structure. All these changes are visible in the array of spectra presented in Figure S2 in the supplementary materials.

## 4. Discussion

This study builds on the recently reported evidence that BPC 157 therapy might resolve very severe vascular failure disturbances, with a particular effect on blood vessels [12,13,14,15,16,17,18,19,20,21,22,23,24,25,26]. As an approach not employed before, we used the FTIR study of rat thoracic aortas, the changes in aortic tissues, and infrared spectroscopy to reveal the particular influence of BPC 157 on vascular tissue components. It might be that these timely in vivo and ex vivo findings correlate with the evidenced functional improvement of vessels. In general, this might indicate that particular changes might lead functionally improved minor vessels to take over the function of disabled major vessels to compensate otherwise irreparable vascular failure, i.e., vascular occlusion [12,13,14,15,16,17,18,19,20,26], compression [21], bile duct occlusion [25], or drug-induced vascular damage [22,23,24]. Based on this FTIR study, this therapeutic effect appeared to be complex. Thus, it might involve both changes in the lipid contents and in protein secondary structure conformation.

In general, the FTIR results showing significant differences between the BPC 157-treated and control group samples in the spectral region between 1450 and 1800 cm^−1^ (part of the spectrum below 1400 cm^−1^ was not reliable because it was under the strong influence of baseline correction) might be highly reliable. At this point, we used a PCA assessment to overlap the FTIR spectra of the biological samples, as they are often extremely similar and usually only advanced chemometric methods can reveal the possible distinctions between different pathological states.

We found that the amid I band around 1650 cm^−1^, the amid II band around 1540 cm^−1^, and the vibrational band around 1744 cm^−1^ were the main bands affecting the separation of the BPC 157-treated and control sample spectra. The amide II and amide I bands originated from the vibrations of the amide groups (CO-NH) of proteins [39], and the vibrational band at 1744 cm^−1^ corresponded to the non-hydrogen-bonded ester carbonyl C=O stretching mode within phospholipids. There were also smaller changes in the range between 2840 and 3090 cm^−1^. This spectral range belonged to the symmetric C-H and asymmetric C-H, C-H_2_, and C-H_3_ vibrations in lipids [36] (Table 2).

The vibrational band at 1744 cm^−1^, much more intense in the control group spectra, could be related to dying cells [40,41] (note that the period between sacrifice and FTIR assessment was 90 min). Moreover, we did not observe any significant change in other lipid vibrational bands, so the increase in the band at 1744 cm^−1^ was not caused by a simple increase in the number of lipid molecules [40]. The fact that the peak at 1744 cm^−1^ was significantly more intense than the peak at 1725 cm^−1^ could also have been related to cell death [40], as it implied that the C=O ester carbonyl groups of lipids in the cell had become predominantly non-hydrogen-bonded, suggesting that oxidative damage had occurred [40]. Therefore, the recorded spectra suggested that the tissue we observed was in an early stage of cell death.

Along with the above evidence (i.e., cells in control samples were in an early stage of cell death), there were also changes in protein conformation. The average position of the amid II band in the control group was shifted toward lower wavenumbers, and the amid I vibrational band had to be fitted with two Gaussians instead of one, as in the case of BPC 157-treated samples. The intensity values of the amid I and amid II vibrational bands were much lower in the control group spectra. Thus, the cells in BPC 157-treated samples were still protected.

Of note, in line with these specific findings on the vascular wall components, to resolve this interconnected entirety, it has been proposed that BPC 157 therapy might modulate vasomotor tone [5,42,43,44,45]. Obviously, it might exert the described particular effects, given the relaxation noted in the aorta with endothelium as well as the relaxation noted in the aorta without endothelium ex vivo [44]. Possibly, these findings together might suggest additional particular points along with the evidenced release of NO on its own [5,42,43,44,45], such as the activated phosphorilazation of eNOS [44], the counteraction of the adverse effect of the NOS blockade (i.e., L-NAME hypertension and pro-thrombotic effect) [8,42], and the counteraction of the adverse effect of NOS over-stimulation (i.e., L-arginine hypertension and anti-thrombotic effect) [8,42]. The VEGFR2-Akt-eNOS signaling pathway might be activated without the need for other known ligands or shear stress, controlling vasomotor tone and the activation of the Src-Caveolin-1-eNOS pathway [44,45]. These might also be perceived as BPC 157-specific mechanisms, in addition to direct NO stimulation.

This clarification might be important, as multiorgan failure, progressive thrombosis, ECG disturbances, blood pressure disturbances (i.e., intracranial (superior sagittal sinus), portal and caval hypertension and aortic hypotension), and commonly progressing Virchow triad might all be compensated by rapidly activated alternative (or collateral) pathway(s) [12,13,14,15,16,17,18,19,20,21,22,23,24,25,26]. The importance of these cytoprotective effects needs to be additionally substantiated [4,5]; this is the first study to investigate the characteristics of removed aortic tissues under the influence of a drug in terms of the detailed specification of direct cell protection assessed with infrared spectroscopy.

This should be also reviewed with respect to the maintenance of thrombocyte function (i.e., without interfering with coagulation pathways) [7,8,9], as well as BPC 157 interaction with non-steroidal anti-inflammatory drugs (NSAIDs); the counteraction of all adverse effects, both non-selective and selective [7,9,10,46,47,48,49,50,51]; and prostaglandin system recovery [6]. In addition, BPC 157 acts as a membrane stabilizer (counteracting leaky gut) [10] and a free radical scavenger, particularly in vascular studies [10,11,12,13,14,15,16,17,18]. Finally, these effects are also associated with several other molecular pathways [10,11,12,32,52,53,54,55,56,57].

In this study, within the frame of FTIR studies, the results revealed that all the particular parts of this recovering effect (i.e., change in the lipid contents and protein secondary structure conformation) might indeed occur rapidly, within a short time from application. These processes in BPC 157 therapy [1,2,3,4,5,12,13,14,15,16,17,18,19,20,21,22,23,24,25,26] might act directly on blood vessels, and they might provide an effective range for prolonged vessel function survival under the very harmful conditions that might occur during vessel removal.

## Figures and Tables

**Table 1:** Assignment of some relevant vibrational bands and components [36,37,38,39].

Wavenumber (cm^−1^)	Vibrational Mode Assignment
∼2960	ν_as_CH_3_ of lipids, proteins, and nucleic acids
∼2924	ν_as_CH_2_ of lipids
∼2874	ν_s_CH_3_ of lipids, proteins, and nucleic acids
∼2853	ν_s_CH_2_ of lipids
∼1744	ester carbonyl C=O stretching mode
∼1654	amide I of proteins
∼1625	β-sheet of amide I
∼1641	Random coil of amide I
∼1660	α-helix of amide I
∼1683	turn of amide I
∼1540	amid II of proteins
aν = stretching vibrations, s = symmetric vibrations, and as = asymmetric vibrations.	

**Table 2:** Initial fitting parameters. Four Gaussians for the control group and three Gaussians for the BPC-treated group, with maximal number of iterations = 200 and tolerance = 10^−6^.

Control Group					
Peak Pos. (cm^−1^)	Bounds (cm^−1^)	FWHM (cm^−1^)	Bounds (cm^−1^)	Intensity (a.u.)	Bonds
1535	free	110	free	0.8	free
1645	±15	55	±15	0.8	free
1685	±15	55	±15	0.8	free
1744	free	30	free	0.8	free
**BPC-Treated Group**					
1535	free	100	free	3	free
1650	free	70	free	3	free
1744	±5	20	±10	0.15	free

**Table 3:** Average vibrational band parameters obtained by fitting.

	Control (cm^−1^)				BPC 157-Treated (cm^−1^)		
Position	1743.7 ± 0.8	1689 ± 1	1644 ± 1	1539 ± 2	1744.4 ± 0.8	1653.7 ± 0.7	1543.4 ± 0.5
Intensity	1.08 ± 0.12	0.90 ± 0,22	1.2 ± 0.2	1.3 ± 0.2	0.27 ± 0,05	3.8 ± 0.4	3.1 ± 0.2
FWHM	34 ± 2	50.0 ± 0.1	64 ± 2	112 ± 6	18 ± 1	74 ± 1	99 ± 4

## Data Availability

The data presented in this study are available upon request from the corresponding author.

## Supplementary Materials

The following supporting information can be downloaded at: https://www.mdpi.com/article/10.3390/biomedicines10123130/s1, Figure S1: Example of two raw spectra: BPC treated and control group; Figure S2: Spectra of BPC treated and control samples after preprocessing.
