# Supplementary material for: Fourier Transform Infrared Spectroscopy Reveals Molecular Changes in Blood Vessels of Rats Treated with Pentadecapeptide BPC 157
Source: Biomedicines. 2022 Dec 4;10(12):3130. doi: 10.3390/biomedicines10123130 (PMC9775416; doi:10.3390/biomedicines10123130)
Supplement: Supplementary file 1 [file biomedicines-10-03130-s001.zip › biomedicines-1989730-supplementary.pdf]

Supplementary materials

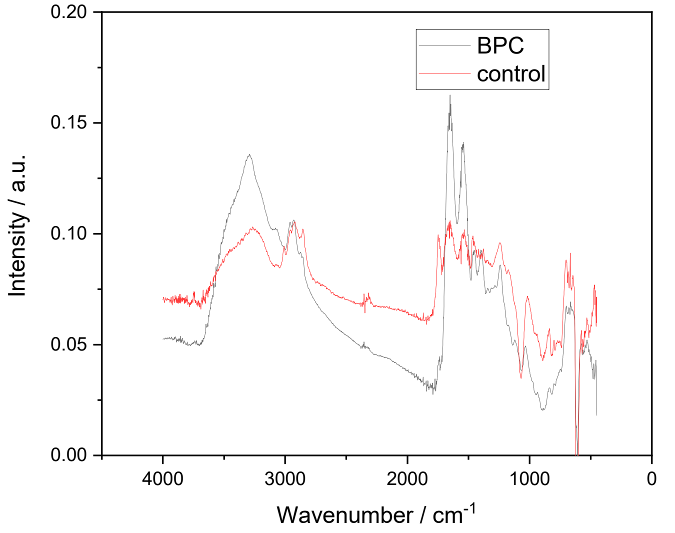

Figure S1. Example of two raw spectra: BPC treated and control group

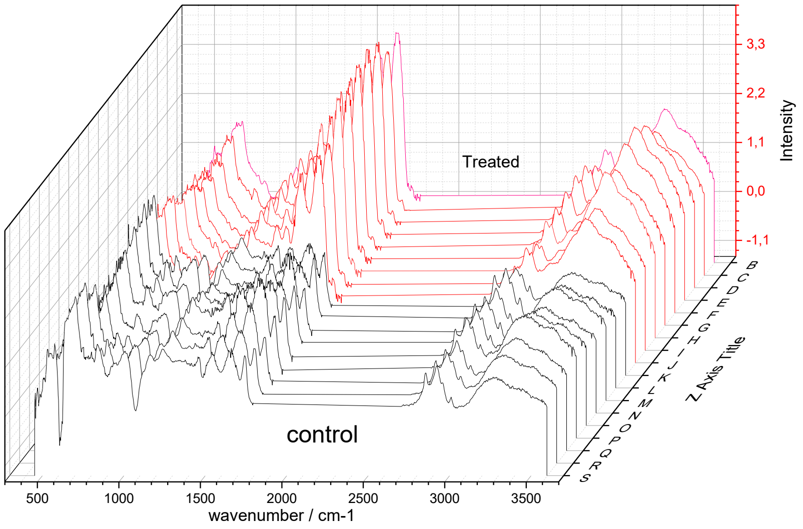

Figure S2. Spectra of BPC treated and control samples after preprocessing
